# Antioxidant, Immunomodulating, and Microbial-Modulating Activities of the Sustainable and Ecofriendly* Spirulina*

**DOI:** 10.1155/2017/3247528

**Published:** 2017-01-15

**Authors:** Alberto Finamore, Maura Palmery, Sarra Bensehaila, Ilaria Peluso

**Affiliations:** ^1^Center of Nutrition, Council for Agricultural Research and Economics (CREA-NUT), Via Ardeatina 546, 00178 Rome, Italy; ^2^Department of Physiology and Pharmacology “V. Erspamer”, “Sapienza” University of Rome, Rome, Italy; ^3^Laboratory of Natural and Local Bioresources, Department of Biology, Faculty of Sciences, University of Hassiba Benbouali, 02000 Chlef, Algeria

## Abstract

The highly nutritional and ecofriendly* Spirulina* (*Arthrospira platensis*) has hypolipidemic, hypoglycemic, and antihypertensive properties.* Spirulina* contains functional compounds, such as phenolics, phycocyanins, and polysaccharides, with antioxidant, anti-inflammatory, and immunostimulating effects. Studies conducted on* Spirulina* suggest that it is safe in healthy subjects, but attitude to eating probably affects the acceptability of* Spirulina* containing foods. Although the antioxidant effect of* Spirulina* is confirmed by the intervention studies, the concerted modulation of antioxidant and inflammatory responses, suggested by in vitro and animal studies, requires more confirmation in humans.* Spirulina* supplements seem to affect more effectively the innate immunity, promoting the activity of natural killer cells. The effects on cytokines and on lymphocytes' proliferation depend on age, gender, and body weight differences. In this context, ageing and obesity are both associated with chronic low grade inflammation, immune impairment, and intestinal dysbiosis. Microbial-modulating activities have been reported in vitro, suggesting that the association of* Spirulina* and probiotics could represent a new strategy to improve the growth of beneficial intestinal microbiota. Although* Spirulina* might represent a functional food with potential beneficial effects on human health, the human interventions used only supplements. Therefore, the effect of food containing* Spirulina* should be evaluated in the future.

## 1. Introduction

*Spirulina* (*Arthrospira platensis*) is a microscopic and filamentous cyanobacterium that has been suggested as a sustainable and ecofriendly microalga useful for bioremediation, nitrification, and carbon dioxide (CO_2_) fixation. In the context of the bioremediation,* Spirulina* is considered a candidate for the removal of toxicants, such as heavy metals [1–5] and phenol [6]. Besides, within microalgae and cyanobacteria* Spirulina* showed maximum biomass productivity with the highest CO_2_ fixation rate [7] and it has been suggested for the nitrification from urine in urban wastewaters [8]. The environmentally friendly* Spirulina* does not need fertile land, has a rapid growth, and takes less energy input and less water per kilogram than soya and corn proteins [9]. Due to its cost-effective and high nutritional value* Spirulina* has been used as protein-rich animal feed for improving meat production and quality [10] and has been proposed as a sustainable approach to prevent Protein Energy Malnutrition (PEM) and Protein Energy Wasting (PEW) in humans [9].

On the other hand,* Spirulina* presents hypolipidemic [11], hypoglycemic [12], and antihypertensive [13] properties. Studies in rats suggested that* Spirulina* increases the lipoprotein lipase activity [14] and the pancreatic secretion of insulin [15]. The latter effect was observed also in mice treated with phycocyanin isolated from* Spirulina* and was accompanied by a decrease in cholesterol, triglycerides, and malondialdehyde (MDA) and by an increase in the serum total antioxidant capacity [16]. On the other hand, the oral administration of an antihypertensive peptide, purified by* Spirulina* and resistant to in vitro digestion by gastrointestinal proteases, decreased both systolic (SBP) and diastolic (DBP) blood pressure in spontaneously hypertensive rats [17].

All these effects could be considered useful in the prevention of the metabolic syndrome. In fact, according to the World Health Organization (WHO), high serum triglyceride level, low serum high-density lipoprotein (HDL) cholesterol level, hypertension, and elevated fasting blood glucose are four of the five risk factors (three out of the five required) for the diagnosis of metabolic syndrome [18]. Metabolic syndrome is associated with subclinical low grade inflammation, oxidative stress, and intestinal dysbiosis and it has been suggested that the gut microbiota could be a target for nutraceuticals [18]. In this regard, in vitro antimicrobial activity [19–21] and the capacity to improve the growth of probiotics [22–24] have been reported for* Spirulina*.

Furthermore,* Spirulina* contains many functional bioactive ingredients with antioxidant and anti-inflammatory activities, including phenolic phytochemicals [25, 26] and the phycobiliprotein C-phycocyanin [27].

We aimed to evaluate the possibility that* Spirulina* could be an antioxidant and immunomodulating functional food by reviewing the human evidences, after taking into account safety and acceptability aspects.

## 2. Functional Compounds of* Spirulina*

*Spirulina* has high nutritional values due to its content in proteins, essential amino acids, minerals, essential fatty acids, vitamins, and liposoluble antioxidants (vitamin E and carotenoids) [28–38] (Table 1).

Great attention has been given to* Spirulina* antioxidant and anti-inflammatory activities in many animal species [16, 34, 38–50] that could not be explained only by* Spirulina* macro and micronutrient content (Table 1).

It has been suggested that the antioxidant activity accounts for the protective role of* Spirulina* against the toxicity induced by carbon tetrachloride (CCl4) [40], by metals (arsenic, mercuric chloride, chromium, cadmium, and fluoride) [34, 41–44], by the insecticide deltamethrin in mice [39] and rats [45], and by the drugs tilmicosin (in mice) [46], gentamicin (in rats) [47], and erythromycin in Egyptian Baladi bucks (*Capra hircus*) [38].

Furthermore, antioxidant effects of* Spirulina* have been reported also in murine models of inflammation [48–50]. In rat models of experimental colitis (acetic-acid induced) [48] and arthritis (Freund's adjuvant-induced [49] and collagen-induced [50]) the antioxidant activity of* Spirulina* was associated with anti-inflammatory effects. Abdel-Daim et al. [39] recently observed a decrease of the proinflammatory cytokine tumor necrosis factor-alpha (TNF-*α*) in serum and at the same time an improvement of oxidative stress markers [malondialdehyde (MDA), nitric oxide (NO), superoxide dismutase (SOD), catalase (CAT), reduced glutathione (GSH), and glutathione peroxidase (GPX)] in hepatic, renal, and brain tissues, by using* Spirulina platensis* powder (500 and 1000 mg/kg) 1 h before deltamethrin (15 mg/kg) in mice [39].

Hu et al. [51] suggested a potential concerted modulation of nuclear factor-erythroid 2-related factor 2 (Nfr2)/antioxidant responsive elements (ARE) and nuclear factor-kappa B (NF-kB) in inflammation and carcinogenesis. Many phenolic antioxidants [52, 53] and C-phycocyanin [54, 55] exert their anti-inflammatory and antioxidant effects through the integrated modulation of Nrf2 and NF-kB pathways. In particular, C-phycocyanin was able to inhibit NF-kB [54] and induced Nrf2 activation in pancreatic *β*-cell INS-1 [55]. Although* Spirulina* antioxidant and anti-inflammatory activities can be due to both phenolic compounds and phycocyanins, C-phycocyanin is contained in higher amounts (Table 1) and has been studied more in vitro [56–63] and in animal models [27, 57, 64–66]. In addition to the scavenging property of C-phycocyanin [56, 57], in cellular models it exerted the antioxidant activity also regulating the antioxidant enzymes activity, such as SOD, CAT, and GPX [58] and inhibiting the cyclooxygenase-2 (COX-2) [63] and the inducible nitric oxide synthase (iNOS) gene expression [63]. Furthermore, a 50% inhibitory concentration (IC50) of 180 nM has been found in a COX-2 isolated enzyme assay [62]. The effects of C-phycocyanin on COX-2 [64, 65] and iNOS [64] were also confirmed in animals, where a decrease of the proinflammatory TNF-*α* expression in the carragenan-induced rat paw [64] and of the TNF-*α* and interleukin-1*β* (IL-1*β*) expression in the cochlea and inferior colliculus after salicylate-induced tinnitus in mice was observed [65]. Also* Spirulina* lipid extract [67] has been shown to repress proinflammatory cytokine (TNF-*α*, IL-1*β*, and IL-6) expression and secretion* via* inhibition of NF-kB pathway. Besides,* Spirulina* contains also heptadecane, a volatile component which has been shown to suppress proinflammatory gene expressions by reducing NF-kB activity [68]. However, it must be taken into account that polysaccharides [69] contained in* Spirulina* (Table 1) can induce NF-kB pathway. In particular, the high molecular weight polysaccharide Immulina has been reported have immunostimulatory activity [70–72] and increased IL-1*β* and TNF-*α* expression by inducing NF-kappa B pathway [72]. Despite the fact that the immunostimulatory activity could increase inflammation, it must be taken into account that in syngeneic tumor-implant mice (C57BL/6 versus B16 melanoma) the antitumor activity and the increased NK cytotoxicity were observed in parallel with the production of interferon-gamma (IFN-*γ*) [73]. Furthermore, it was also observed that* Spirulina* consumption increased macrophage activation (phagocytic activity and nitrite production) in chicken [74]. Therefore,* Spirulina* could modulate immune function, reducing inflammation without inhibiting the innate immune defences.

## 3. Microbial-Modulating Activities

It has been recently reported that, in the majority of commercially available* Spirulina* food supplements,* Arthrospira platensis* was the predominant taxon (81.2–100.0%) among the cyanobacteria [75].

**Figure 1:**
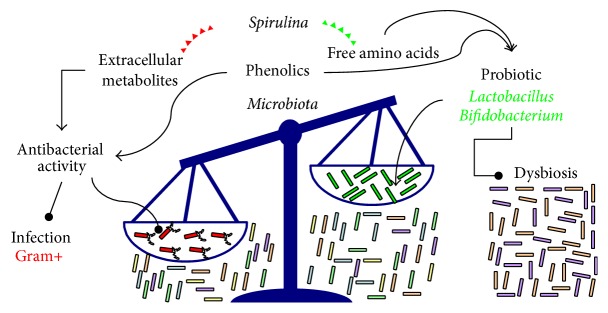
Microbial-modulating activities of* Spirulina* could prevent dysbiosis. The antibacterial activity of Spirulina could protect the host by infections. Changes in microbiota composition are commonly associated with several several diseases including inflammatory bowel diseases (IBD) and metabolic and immunological diseases. Alterations of gut microbial composition also result in changes in the metabolites generated in the gut from microbial activity, essential for a correct gut homeoastasis.* Spirulina* intake may favour a restabilishment of correct microbial balance by promoting probiotic species growth.

*Spirulina (Arthrospira) platensis* is able to inhibit the growth of some Gram-negative* (Escherichia coli, Pseudomonas aeruginosa,* and* Proteus vulgaris)* and Gram-positive bacteria* (Staphylococcus aureus, Bacillus subtilis,* and* Bacillus pumulis)* [76]. In fact,* Spirulina* produces extracellular metabolites with antibacterial activity (Figure 1) [19–21, 77]. The methanol extract from grown culture medium of* Spirulina* showed a higher antimicrobial activity than hexane [21], dichloromethane [20, 21], petroleum ether [20], ethyl acetate [20, 21] extracts, and volatile components (heptadecane and tetradecane) [20], especially against* Streptococcus faecalis* [20],* Staphylococcus epidermidis* [20] and* Candida albicans* [20], Gram-positive bacterium* Staphylococcus aureus* [21], and Gram-negative bacterium* Escherichia coli* [21].

On the contrary, low (minimum inhibitory concentrations, MIC ≥ 512 *μ*g/ml) or no inhibitory effect was found against other bacteria (*Pseudomonas aeruginosa*,* Salmonella typhirium*, and* Klebsiella pneumoniae*) [21]. El-Sheekh et al. [19] purified an antimicrobial compound (molecular formula C_15_H_18_NO_8_) from* Spirulina platensis* with no characteristic odor and yellowish green color. This extract (soluble in methanol, diethyl ether, chloroform, and dimethyl sulfoxide, but sparingly soluble in water and acetone) was active against the unicellular fungus* Candida albicans* (MIC = 30 *μ*g/ml) and the Gram-positive* Bacillus subtilis* (MIC = 60 *μ*g/ml) at lower concentrations in comparison to the effect against the Gram-negative bacterium* Pseudomonas aeruginosa* (MIC = 85 *μ*g/ml) [19]. Besides,* Spirulina* has been recently used in the synthesis of biofunctionalized gold nanoparticles with antibacterial activity against Gram-positive organisms (*Bacillus subtilis* and* Staphylococcus aureus*) [77]. Therefore, the research on advanced medical applications of* Spirulina*-derived products in the treatment of infectious diseases caused by Gram-positive organisms is growing (Figure 1).

On the other hand, it has also been reported that extracellular products of* Spirulina*, obtained from a culture in late exponential stage and separated by filtration, significantly promote the in vitro growth of the lactic acid bacteria* (Lactococcus lactis*,* Streptococcus thermophilus*,* Lactobacillus casei*,* Lactobacillus acidophilus*, and* Lactobacillus bulgaricus*) [22]. Probiotics, including the genera* Lactobacillus* and* Bifidobacterium* (Figure 1) [78], are largely used as starter bacteria for the production of yogurt [24], the most popular fermented diary product worldwide.* Spirulina* biomass has a stimulatory effect on the growth (during fermentation) and/or increases the survival (during storage) of* Bifidobacterium* [23, 24],* Lactobacillus acidophilus* [24, 76],* Lactobacillus bulgaricus* [79–81],* Lactobacillus casei* [76], and* Streptococcus thermophilus* [23, 76, 79, 80, 82].

Although the better growth and survival have been attributed to the high level of nitrogenous substances, in particular free amino acids, in the* Spirulina* biomass [109], also phenolic compounds have been shown to exert antimicrobial or bacteriostatic activities, as well as improving the growth of probiotics [18]. Therefore, the complex composition of* Spirulina* could improve the quality of fermented diary products and the supplementation with* Spirulina* might represent an alternative strategy to the synbiotics formulations. The latter appear more effective than probiotics alone in the prevention of the dysbiosis (Figure 1) associated with immune-mediated, inflammatory, and dysmetabolic diseases [78].

Despite the rich literature on in vitro effect of* Spirulina*, only few studies have been conducted in vivo. Although in mice the gut microbiota of the* Spirulina*-fed group was 70% similar to that of the control mice [110], changes of gut microbiota ecology induced by* Spirulina *feeding in mice have been showed by Rasmussen and collegues [110]. The authors observed that* Spirulina plantensis* leads change to gut microbiota composition in mice reducing* Bifidobacterium animalis* and increasing* Clostridium irregulare*, suggesting that numbers of this organism are modified through both blue-green algae supplements [110]. It is largely established that drastic changes of microbiota composition occur in several gastrointestinal, immunological, and metabolic diseases [111, 112]. In many microbiota related diseases, including Inflammatory Bowel Disease (IBD), it is well known that a strong unbalanced ratio among the genera of potentially protective bacteria and normal anaerobic bacteria is present. In particular,* Bacteroides* sp.,* Eubacterium* sp., and* Lactobacillus* sp are significantly decreased [113]. All these evidences suggest that* Spirulina* may be useful to improve animal and human health changing the gut microbiota composition and promoting beneficial batcterial growth.

## 4. Safety and Acceptability

The Food and Drug Administration (FDA) has categorized* Arthrospira* products as “generally recognized as safe” (GRAS) for human consumption and the Dietary Supplements Information Expert Committee (DSI-EC) concluded that there is not a serious risk to health with consumption of* Spirulina* [114].

Reported side effects associated with* Spirulina* consumption are insomnia and gastric problems with uncertain or unlikely causality [114] and only few cases of severe side effects have been reported, including a case of rhabdomyolysis after the consumption of 3 g/day for 1 month [115]. Two cases of anaphylaxis caused by* Spirulina* tablets were reported [116, 117] of which one in a 17-year-old male who had a history of atopic dermatitis, asthma, allergic rhinitis, and a possible pollen-food syndrome (oral allergy symptoms to tomato and cucumber) [117]. Three cases of autoimmune-mediated skin damage were reported, of which one in a 82-year-old woman [118], whereas the other two cases were observed in subjects consuming* Spirulina* as ingredient of multicomponent-nutraceuticals (organic cayenne pepper, ethylsulfonylmethane, and the algae* Aphanizomenon flos-aquae* and* Spirulina* or Ginseng, Ginkgo biloba, and* Spirulina*) [119]. In this regard, it is well known that plant-food and herbal supplements could have adverse effects, such as hepatotoxicity and autoimmune hepatitis [120]. Besides, the two cases described by Lee and Werth [119] involved a 57-year-old man with known pemphigus vulgaris and a 45-year-old woman with a history of hypertension, chronic migraines and fibromyalgia. Furthermore, a case report of hepatotoxicity involved a 52-year-old Japanese man who had a history of hypertension, hyperlipidemia and type 2 diabetes (T2D) and taking medications (amlodipine besilate, simvastatin, and acarbose) [121]. In this context, potential food-drug interactions have been hypothesized for* Spirulina* [122] and for phenolic phytochemicals [123–125]. Therefore,* Spirulina* should be ingested with caution in subjects with diseases, in particular in patients in treatment with substrates of cytochrome P450 enzymes, such as immunosuppressant, antihypertensive, and lipid lowering drugs [121–125].

Although* Spirulina* can be considered safe in healthy subjects, sensory characteristics of a functional food are important in the consumer acceptance of the product.

Table 2 shows the studies that have investigated the overall acceptability of foods with* Spirulina*, including baby formulas, pomegranate juices, biscuits, snacks, pasta, ice creams, yogurt, and acidophilus milks [32, 36, 85–92]. The number of panelists ranged between 4 and 43, and the point scale was different between studies (Table 2). Only a study on functional biscuits containing* Spirulina* or phycocyanin isolated from* Spirulina* reported similar levels of acceptability versus control [36]. In the other studies, both higher and lower levels of acceptability were reported (Table 2). The results were affected by the type of product, by the percentage of* Spirulina* and by the type of panelists.

Baby food formulas with added* Spirulina* had an overall acceptability scores in the range from 82.72 to 96.37 and the trained panelists assigned the high scores to products with* Spirulina* 5% [32].

No significant differences were noticed by semitrained panelists between the pomegranate juices and formulate pomegranate beverage with* Spirulina* (4%) and Echinacea (6%) extracts (sweetened by stevioside 5%) in appearance, color, odors, and consistency, while the other parameters including taste and overall acceptability showed a significant decrease in the mean values of pomegranate juice compared to the fresh formulate pomegranate beverage [85].

Trained panelists gave a higher score to a snack with 2.5% of* Spirulina*, but the addition of 7.5% or more decreased the acceptability [86]. The percentage of* Spirulina* in pasta considered acceptable is different in trained and untrained panelists. In particular, pasta with a percentage of* Spirulina maxima* up to 2% was preferred by untrained panelists compared to control pasta [87], whereas the most preferable one by trained panelists [88] was the pasta enriched with 10%* Spirulina platensis*. The latter was not acceptable for consumers [89] who considered less acceptable also pasta produced with integral wheat flour. The percentage of* Spirulina* that did not decrease the acceptability is lower for ice cream compared with supplemented pasta (Table 2). A panel of judges considered ice cream with 0.15% of* Spirulina* a superior product when compared to 0% and 0.075% ice creams, due to the light green (pistachio) color, but the intense green color decreased overall acceptability of the ice cream with 0.23% and 0.3% of* Spirulina* [90]. On the contrary, yogurt with* Spirulina* 0.3% had a higher score compared to 0%, 0.1%, 0.2, and 0.5% of* Spirulina* [91]. Malik et al. [91] suggested that the lower score at 0.1% level when compared to control may be attributed to dull color and appearance and less acidic flavor which is essential for acceptability of yogurt, whereas the low acceptability of the 0.5% can be due to increased acidity and intense green color. It is well known that* Spirulina* causes decrease in pH of yogurts, due to its effect on Lactobacilli growth and viability [82, 126]. Guldas and Irkin [92], with trained panelists who did not assign excessive sour taste to acidophilic yogurt, reported that the 0.5% of* Spirulina* powder addition was more acceptable than 1%, due to the slightly greenish color and algal flavor of the latter compared to the former. Therefore, different sensory expectations, experiences, knowledge, learning, and attitude to eat affect the overall acceptability [127, 128].

## 5. From Nutritional Supplement to Antioxidant and Immunomodulating Functional Food in Humans

Due to its high nutritional value,* Spirulina* has been used for treatment of anemia and malnutrition in undernourished children [28, 29, 129] and disease patients [29, 103, 130, 131]. Positive effects of* Spirulina* at doses ranging between 1 g/day [131] and 200 g/day [28] against anemia have been reported in children [28, 29, 129], runners [132], senior citizens [133], patients with pathologies such as nonalcoholic fatty liver disease (NAFLD) [130], T2D [131], or HIV-infected [29, 103]. Only two studies did not find improvement in Hemoglobin (Hb) levels after* Spirulina* supplementation. In the first study (*Spirulina* 6 g/day, 30 days), Hb was measured only in a subgroup of 5 subjects, probably not enough to reach statistical significance [101]. In the second study, both supplemented and control groups received also dietary products supplied by the World Food Program (WFP) and showed improvement of protidemia [104]. Besides in HIV-infected patients [29, 103] and undernourished children [28, 29] increases in body weight were reported, probably due to protein content. In agreement with this hypothesis, in HIV-infected antiretroviral-naïve patients an increase in fat free mass (FFM) has been observed versus soya supplementation [103]. On the other hand,* Spirulina* did not affect body weight in subjects with dyslipidemia [134]. In this context,* Spirulina* reduced plasma lipids concentrations in many studies [12, 13, 100, 106, 108, 130, 134–137]. Accordingly, the results of a recent meta-analysis [11] of 7 Randomized Controlled Trials (RCT) showed a significant effect of supplementation with* Spirulina* in reducing plasma concentrations of total cholesterol (−46.76 mg/dL, *p* < 0.001), low density lipoprotein (LDL) cholesterol (−41.32 mg/dL, *p* < 0.001), and triglycerides (−44.23 mg/dL, *p* < 0.001) and elevating those of HDL cholesterol (+6.06 mg/dL, *p* = 0.001). In overweight subjects [13] and in T2D patients with dyslipidemia [106] the improvement in the lipid profile was accompanied by a reduction of blood pressure. Furthermore, some studies found also decreased levels of glucose or HOMA-IR after* Spirulina* supplementation in healthy volunteers [12], in subjects with NAFLD [130], T2D [138], and HIV-infected antiretroviral-naïve patients [102, 135].

Table 3 summarizes the 17 studies [29, 93–108] that investigated the effect of long term consumption (duration range: 7 days [96]–12 months [102]) of* Spirulina* or* Spirulina*-derived Immulina on markers of immune and redox status.

The majority of the studies had a longitudinal (uncontrolled) or parallel design (controlled or uncontrolled), and only two studies followed a crossover design. The number of participants in individual trials was extremely variable, ranging from 8 [100] to 169 [102], and characteristics of subjects varied between studies. In particular, healthy subjects, elderly, runners, children, patients with HIV infection, and T2D patients with allergic rhinitis or chronic obstructive pulmonary disease (COPD) were enrolled (Table 3).

Various biomarkers were used to monitor different aspects of redox and immune status in biological fluids and cells. Markers of redox status included total antioxidant status (TAS, *n* = 4), vitamin C (vit. C, *n* = 2), GSH (*n* = 2), antioxidant enzymes (*n* = 4) (*e.g.,* SOD, CAT, and GPX), and markers of lipid peroxidation (*n* = 7) [*e.g.,* MDA, thiobarbituric acid reactive substances (TBARS), and peroxides (ROOH)]. Markers of immune function included cytokines (*n* = 3), ex vivo cytokines' production by peripheral blood mononuclear cells (PBMC) (*n* = 3), lymphocytes' populations (*n* = 7), lymphocytes' proliferation (*n* = 1), and NK cytotoxic activity (*n* = 2).

Unchanged NK, NKT, and T cells were found after* Spirulina* consumption in healthy subjects [96]. Increases in T helper lymphocytes (CD4+ counts) were reported in HIV-infected patients (Table 3). In particular, the increase in CD4+ count was always accompanied by a decrease in the viral load [101–103]. This effect could be mediated by the antiviral activity against HIV of the natural sulfated polysaccharide (calcium spirulan), contained in* Spirulina* [139]. Accordingly, the increase in lymphocytes was observed in HIV-positive, but not in HIV-negative undernourished children [29]. Furthermore, Winter et al. [105] reported no effects on viral load with consequent progression of disease (*e.g.,* decrease of CD4+ cells), in HIV patients, despite the increase in TAS. Also other three studies measured both redox and immune markers [98, 99, 106].

A recent study in elderly found a lower increase in the IL-2/IL-6 ratio in obese compared to normal weight (NW) after* Spirulina* supplementation [98]. Furthermore, only in NW subjects an improvement of TAS and TBARS levels after treatment was observed [98]. Also gender differences were found in elderly after* Spirulina* consumption, with increase of IL-2 and SOD in female and decrease of IL-6 accompanied by increase in TAS in males [99]. The third study, conducted in T2D patients, found decreased MDA levels that were associated with decrease of IL-6 only in patients with dyslipidaemia [106]. Therefore, the concerted modulation of redox and inflammatory status by* Spirulina* in humans requires more studies.

On the other hand, improvement of at least one marker of redox status (decrease in markers of peroxidation and/or increase in antioxidant enzymes, TAS, GSH, or vitamin C) was reported in healthy subjects (7.5 g/day, 3 weeks) [95], elderly (8 g/day, 12 and 16 weeks) [98, 99], runners (4 g/day, 2 weeks) [100], COPD (1 g and 2 g/day, 60 days) [108], and T2D (8 g/day, 12 weeks) [106] patients. Only Shyam et al. [97] reported decreased GSH and unchanged MDA, TAS, vitamin C, and SOD in healthy subjects after* Spirulina* (1 g/day, 30 days).

Concerning the ex vivo markers of immune function, NK cytotoxic activity increased after both 7 days [96] and 8 weeks [94] of* Spirulina* or* Spirulina*-derived Immulina supplementation, whereas data on proliferation of lymphocytes and cytokines' production varied with the stimulus used (BCG-CWS: cell wall skeleton of* Mycobacterium bovis* Bacillus Calmette-Guérin; CA:* Candida albicans*; Con A: concanavalin A; PHA: phytohemagglutinin; or TT: tetanus toxoid) and the duration of supplementation (Table 3), also in the same study [93]. In particular, after the* Spirulina*-derived Immulina 400 mg/day for 56 days, a significant correlation between age and the increase in TT-induced CD4+ proliferation was found, while significant correlations were not found with respect to CA response. On the other hand, no effect was observed on TT-induced proliferation of CD19+, whereas the CA-induced CD19+ proliferation was increased after 3–8 days but decreased at day 56. Also the effect on the production of cytokines was temporary. The CA- and TT-induced production of IL-5 a Th2-related cytokine was increased at the beginning of the supplementation (3–8 days) but was inhibited at 56 days. On the contrary, the CA- and TT-induced production of IL-4 was decreased at day 3 and no effect was observed on IL-10 response during all the supplementation period. Concerning Th1 cytokines, after 3, 8, and/or 14 days of consumption a significant increase was observed of the CA- and/or TT-induced production of TNF-*α* (3–8 days, CA and TT), IFN-*γ* (day 3 CA, 3–14 days TT), and IL-2 (only CA day 3 and day 56), whereas at day 56 the TT-induced productions of TNF-*α* and IFN-*γ* were decreased. Although both Th1 and Th2 improvements were temporary, the increase in IFN-*γ* could account for the increased NK activity observed in other studies with* Spirulina* or* Spirulina*-derived Immulina [94, 96].

Overall,* Spirulina* seems to affect more innate immunity than adaptative immunity, but the immunomodulating activity of* Spirulina* in humans requires further investigations.

Furthermore, in the majority of the studies the effect of* Spirulina (Arthrospira) platensis* or a* Spirulina* not specified species has been investigated and only in two studies* Spirulina maxima* has been used (Table 3). Therefore, more studies are needed in order to evaluate the possible specie-specific effects in humans.

## 6. Conclusion

The concerted modulation of antioxidant and inflammatory responses (by Nrf2 and NF-kB pathways), suggested by in vitro and animal studies (Table 1), requires more confirmation in humans (Table 3). It has been suggested that the temporary priming effect on the responses of peripheral Th1, Th2, and B cells to antigenic stimuli could be related to a proinflammatory effect of Immulina [93]. However, the effects on cytokines and on lymphocytes' proliferation are contrasting, depending on age, gender, and body weight differences. In this context, ageing [140], obesity [141, 142], and metabolic syndrome [142] are associated not only to chronic low grade inflammation, but also to immune impairment (recurrent infection and low vaccine efficacy). Therefore the increased immune response to antigenic stimuli could be protective in elderly [93].

On the other hand,* Spirulina* has hypolipidemic, hypoglycemic, and antihypertensive properties, useful in the prevention of the metabolic syndrome [18]. In this context, the alteration of gut microbiota is common in elderly [140], obese [141], and subjects with the metabolic syndrome [18].

From that, the microbial-modulating activities of* Spirulina* (reported in vitro and in animal models, Figure 1) suggest that the association* Spirulina* and probiotics could represent a new synbiotic, maintaining and/or restoring the homeostasis at level of gut microbiota. Human intervention studies are required for confirmation of this hypothesis.

Furthermore,* Spirulina* improves oxidative stress markers and NK activity in healthy subjects and CD4+ count in HIV+ patients.

However, among bioactive molecules from* Spirulina* (Table 1) only Immulina has been tested in humans (Table 3). Therefore the role of bioactive molecules from* Spirulina* for human applications requires further studies. Moreover, despite the fact that* Spirulina* might represent a functional food with potential beneficial effects on human health, the human interventions used supplements (Table 3). Although the tested doses (1–20 g/day) in these studies (Table 3) were not greater than the maximum acceptable percentage (10%) of* Spirulina* in functional foods (Table 2), no data are available on the efficacy of* Spirulina* containing foods. Therefore, the healthy effect of food containing* Spirulina* should be further evaluated.

Besides, previous studies indicate that some antioxidant and immunological markers are sensitive to stimuli that affect the mood of the individual. In particular, the salivary TAC increased 30 minutes after the vision of a comical video [143] and pleasant emotions increase the salivary IgA and cortisol [144]. In this context different species of* Spirulina*, possibly having different biological effects, showed different acceptability [87, 88]. Therefore, the study of the relationship between liking and markers of antioxidant and immune status should be considered in humans studies.

## Figures and Tables

**Table 1:** Nutritional values and functional compounds of *Spirulina*.

	Content in 100 g	The effects in vitro and in animal models	Ref.
Nutritional values	Proteins 35.4–70.0 g *Amino acids*Glutamate 7.0–7.3 g Leucine 5.9–8.4 g Aspartate 5.2–6.0 g Lysine 2.6–4.6 g Tyrosine 2.6–3.4 g Phenylalanine 2.6–4.1 g Methionine 1.3–2.7 g Fat 4.0–16.0 g *% of total fatty acids*Palmitic 25.8–44.9% Gamma-linoleic 17.1–40.1% Linoleic 11.1–12.0% Oleic 10.1–16.6% Palmitoleic 2.3–3.8% Stearic 1.7–2.2% Carbohydrates 14.0–19.0 g Crude fiber 3.0–7.0 g *Minerals* Potassium 2.0–2.6 g Sodium 1.5–2.2 g Total phosphorus 1.3–2.2 g Iron 273.2–787.0 mg Magnesium 330 Calcium 120–900 mg *Vitamins* B12 5.7–38.5 *μ*g B2 3.0–4.6 mg B6 0.5–0.8 mg Niacin (B3) 13–15 mg Folic acid 0.05–9.92 mg Carotenoids 0.3–2.6 g Tocopherol 0.4–9.8 g		[28–38]

Functional compounds	Total phenol 0.20–1.73 g Flavonoids 0.1–0.9 g	Antioxidant Anti-inflammatory	[25, 26, 83, 84]
	Phycocyanins C-Phycocyanin 13.5–14.8 g Allophycocyanin 2.3 g Phycobiliproteins 1.1 g	Antioxidant Anti-inflammatory	[27, 32, 34, 36, 56–66]
	Polysaccharides 0.2–12.5 g	Immunostimulating	[69–72]

**Table 2:** Acceptability of *Spirulina* products.

Products	Panelists (*n*) (point scale, PS)	Acceptability	Ref.
8 fruit-vegetable (SFV) baby food formulas (i) Puree of banana 30%, potato 10%, carrot 10%, apple 15%, guava 15%, mango 15%, sugar 5%, *Spirulina* 0% (1SFV), 2.5% (2SFV), 5% (3SFV), and 7.5% (4SFV) (ii) Puree of papaya 30%, potato 10%, carrot 10%, apple 15%, guava 15%, mango 15%, sugar 5%, *Spirulina* 0% (5SFV), 2.5% (6SFV), 5% (7SFV), and 7.5% (8SFV)* * 8 cereal-based (SCP) baby food formulas (i) Cereals (wheat 30%, barley 30%), legumes (dried peas 10%, lentils powder 10%), vegetable (dried spinach 10%, dried Cauliflower 10%), *Spirulina* 0% (9SCP), 2.5% (10SCP), 5% (11SCP), and 7.5% (12SCP) (ii) Cereals (rice 30%, barley 30%), legumes (dried peas 10%, lentils powder 10%), vegetable (dried spinach 10%, dried Cauliflower 10%), *Spirulina* 0% (13SCP), 2.5% (14SCP), 5% (15SCP), and 7.5% (16SCP)	Trained (12) (100 PS)	Versus 0%: ↑ (high scores *Spirulina* 5%: 3SFV, 7SFV, 3SCP, and 7SCP)	[32]

Pomegranate juice (0%) or pomegranate juice with *Spirulina platensis* (4%) and Echinacea (6%) extracts sweetened by stevioside (5%)	Semi-trained (10) (10 PS)	Versus 0%: ↑	[85]

Biscuits (0%) or biscuits with *Spirulina platensis* 0.3%, 0.6%, and 0.9% or phycocyanin extracts 3.0%	Untrained (20) (9 PS)	Versus 0%: *↔*	[36]

Snacks: corn flour (coating 6% cheese flavor + 19% palm Olean oil) with *Spirulina* 0%, 2.5%, 5%, 7.5%, 10%, and 12.5%	Trained (nr) (9 PS)	Versus 0%: 2.5%↑, 5%*↔*, 7.5%, 10%, and 12.5%↓	[86]

Pasta with *Spirulina maxima* 0%, 0.5%, 1.0%, and 2.0%	Untrained (43) (5 PS)	Versus 0%: ↑	[87]

Pasta with *Spirulina platensis* 5%, 10%, and 15%	Trained (7) (7 PS)	Versus 0%: *↔*10%; ↓5%; and 15%	[88]

Pasta (i) Special wheat flour with 0%, 5%, and 10% of *Spirulina platensis * (ii) Integral wheat flour with 0%, 5%, and 10% of *Spirulina platensis*	Consumers (nr) (9 PS)	Versus 0%: *↔*5%; ↓10% (special wheat flour versus integral wheat flour: ↓)	[89]

Ice cream 0%, 0.075%, 0.15%, 0.23%, and 0.3% of *Spirulina* to replace 0%, 25%, 50%, 75%, and 100% of stabilizer	Judges (4) (100 PS)	Versus 0%: 0.075%*↔*, 0.15%↑, 0.23%, and 0.3%↓	[90]

Yogurt with 0%, 0.1%, 0.2, 0.3, and 0.5% of *Spirulina*	Judges (4) (100 PS)	versus 0%: 0.1 and 0.5%↓; 0.2%*↔*; 0.3% ↑	[91]

(i) Plain yogurt (only yogurt starters) with 0%, 0.5%, and 1% of *Spirulina platensis * (ii) Probiotic yogurt (*L. acidophilus* + yogurt starter bacteria) with 0%, 0.5%, and 1% of *Spirulina platensis * (iii) Acidophilus milk (only *L. acidophilus.*) with 0%, 0.5%, and 1% *Spirulina platensis*	Trained (5) (5 PS)	*↔* versus 0% 0.5% > 1%.	[92]

SFV = spirulina with fruits and vegetables-based baby food formula; SCP = spirulina with cereals-based baby food formula; *n* = numbers; PS = point scale.

**Table 3:** Human intervention studies measuring markers of immune function or redox status after *Spirulina* supplementation.

Subjects (*n*)	Study design and treatment	Markers of immune function	Markers of redox status	Ref.
Healthy (11)	Longitudinal *Spirulina*-derived Immulina 400 mg/day, 56 days	Proliferation ↓ CA-induced CD19+ (day 56) ↑ CA-induced CD19+ (3–8 days), CD4+ (3–56 days) ↑ TT-induced CD4+ (3–8 days) Cytokines' production ↓ TT- induced TNF-*α*, IFN-*γ*, IL-5 (day 56) ↓ CA and TT- induced IL-4 (day 3) *↔* TT-induced IL-2, IL-12, IL-10 ↑ CA-induced TNF-*α* (3–8 days), IL-2 (day 3 and 56), IFN-*γ* (day 3), IL-6 (3–14 days), IL-5 (3–8 days) ↑ TT-induced TNF-*α* (3–8 days), IFN-*γ* (3–14 days), IL-6 (3–8 days), IL-5 (3–8 days)		[93]

Healthy (12)	Longitudinal 50 ml hot water extract of *Spirulina platensis* extract, 8 weeks	↑ NK cell cytotoxic activity Cytokines' production *↔* Con A-induced IL-12 ↑ BCG-CWS-induced IL-12, ↑ IL-12 and IL12/IL18-induced IFN-*γ*		[94]

Healthy (16)	Parallel (versus soya proteins) *Spirulina platensis* or soya 7.5 g/day, 3 weeks (after exercise)		↑ SOD, GPX ↓ MDA	[95]

Healthy (20)	Crossover (placebo controlled) *Spirulina*-derived Immulina 0.2 g and 0.4 g/day, 7 days	↑ NK cell cytotoxic activity *↔* NK, NKT, T cells		[96]

Healthy (30)	Parallel (placebo controlled) *Spirulina maxima* 1 g/day, 30 days		*↔* MDA, TAS, vit. C, SOD ↓ GSH	[97]

Elderly NW (45) Obese (33)	Parallel (placebo controlled) *Spirulina* 8 g/day, 12 weeks	↑ IL-2 (NW 54.1%, obese 33%) ↓ IL-6 (NW 20%, obese 14.6%) *↔* TNF-*α*	↑ TAS (only NW) ↓ TBARS (only NW)	[98]

Elderly (78)	Parallel (placebo controlled) freeze-dried *Spirulina* 8 g/day, 16 weeks	↑ IL-2 (female) ↓ IL-6 (male)	↑ SOD (female), TAS (male) *↔* GPX, TBARS	[99]

Runners (8)	Longitudinal *Spirulina maxima* capsules 4 g/day + 200 ml/day antioxidants drink^†^, 2 weeks		↓ MDA	[100]

HIV+ (84)	Parallel (versus untreated) *Spirulina platensis* 20 g/day, 8 weeks	↑ lymphocytes		[29]

HIV+ (11)	Parallel (uncontrolled) *Spirulina platensis* capsules 6 g/day, 3 months Undaria 5 g/day, 3 months *Spirulina* capsules 3 g/day + Undaria 2.5 g/day, 3 months	↑ CD4 (*n* = 6) ↓ viral load (*n* = 6)		[101]

HIV+ (169)	Parallel (placebo controlled) *Spirulina platensis* powder 10 g/day, 12 months	↑ CD4 ↓ viral load		[102]

HIV+ (52)	Parallel (uncontrolled) proteins 1.5 g/kg body weight (25% *Spirulina platensis* or soya)	↑ CD4 (both groups, ↑ versus soya) ↓viral load (both groups and ↓ versus soya)		[103]

HIV+ (160)	Parallel (placebo controlled)^*∗*^* * *Spirulina* 10 g/day, 6 months	↑CD4 (both groups)		[104]

HIV+ (73)	Parallel (placebo controlled) *Spirulina platensis* powder 5 g/day, 3 months	↓CD4 (both groups) *↔* viral load, CD38 expression on the CD8	↑ TAS	[105]

T2D (37)	Parallel (placebo controlled) freeze-dried *Spirulina* 8 g/day, 12 weeks	↓ IL-6 (only in patients with dyslipidemia) *↔* TNF-*α*	↓ MDA	[106]

Allergic rhinitis (36)	Crossover (placebo controlled) *Spirulina* capsules 1 g and 2 g/day, 12 weeks	Cytokines' production ↓ PHA-induced IL-4 (only 2 g/day) *↔* PHA-induced IFN-*γ*, IL-2		[107]

COPD (30)	Parallel (uncontrolled: 2 doses) *Spirulina* capsules 1 g and 2 g/day, 60 days		↑ CAT (only 2 g/day), SOD, GST, GSH, vit. C ↓ MDA, ROOH	[108]

*n* = numbers; ^†^antioxidants drink containing *β* Carotene 7600 mcg, vitamin A 400 IU, vitamin E 80 IU, vitamin C- 320 mg, zinc-2.7 mg, and selenium-40 mcg; ^*∗*^both groups received also dietary products supplied by the World Food Program (WFP); BCG-CWS: cell wall skeleton of *Mycobacterium bovis* Bacillus Calmette-Guérin; CA: *Candidaalbicans*; CAT: catalase; Con A: concanavalin A; COPD: chronic obstructive pulmonary disease; GSH: glutathione; IL-: interleukin; MDA: malondialdehyde; PHA: phytohemagglutinin; ROOH: lipid hydroperoxides; SOD: superoxide dismutase; T2D: type 2 diabetes; TAS: total antioxidant status; TT: tetanus toxoid.
